# Long-term treatment outcomes of upadacitinib for Crohn’s disease: a single-center retrospective study

**DOI:** 10.1093/crocol/otag047

**Published:** 2026-05-29

**Authors:** Takahiro Ito, Kohei Matsunaga, Yuya Ohno, Muchir Shi, Atsuo Maemoto

**Affiliations:** Inflammatory Bowel Disease Center, Sapporo Higashi Tokushukai Hospital, Sapporo, Japan; Inflammatory Bowel Disease Center, Sapporo Higashi Tokushukai Hospital, Sapporo, Japan; Inflammatory Bowel Disease Center, Sapporo Higashi Tokushukai Hospital, Sapporo, Japan; Inflammatory Bowel Disease Center, Sapporo Higashi Tokushukai Hospital, Sapporo, Japan; Inflammatory Bowel Disease Center, Sapporo Higashi Tokushukai Hospital, Sapporo, Japan

**Keywords:** Crohn’s disease, upadacitinib, JAK inhibitor, real-world evidence, treatment continuation

## Abstract

**Background:**

Upadacitinib is the sole Janus kinase inhibitor approved for Crohn’s disease in Japan, with its short-term efficacy already known. However, its long-term real-world outcomes remain unclear. This study aimed to evaluate its long-term continuation rates in patients with Crohn’s disease and to identify predictive factors for sustained treatment.

**Methods:**

This single-center retrospective study included 41 patients with Crohn’s disease treated with upadacitinib between July 2023 and April 2025. Continuation rates at 1 and 2 years defined the primary outcomes. Furthermore, predictive factors for treatment discontinuation, predictive scoring system development, upadacitinib’s effects on perianal draining fistulas, endoscopic outcomes, and adverse events were the secondary outcomes.

**Results:**

The cumulative continuation rates were 67% and 63% at 1 and 2 years, respectively. In univariate analysis, factors such as age ≥ 40 years, penetrating disease behavior, prior intestinal resection, small-bowel stoma, prior exposure to ≥3 biologics, low baseline inflammation (C-reactive protein < 0.34 mg/dL and Simple Endoscopic Score for Crohn’s Disease < 8), week 4 hemoglobin < 11.4 g/dL, week 4 albumin < 4.1 g/dL, and 5-aminosalicylic acid nonuse were associated with upadacitinib discontinuation. A predictive scoring system (0-8 points) demonstrated that patients with scores <3 had significantly better continuation rates than those with scores ≥3 (median: 703 vs. 341 days). Within 24 weeks of upadacitinib use, 2 of 5 patients developed perianal fistula closure.

**Conclusions:**

In Crohn’s disease, upadacitinib continuation rates can be predicted using baseline patient characteristics and early biomarkers at week 4.

## Graphical abstract

**Graphical Abstract:**
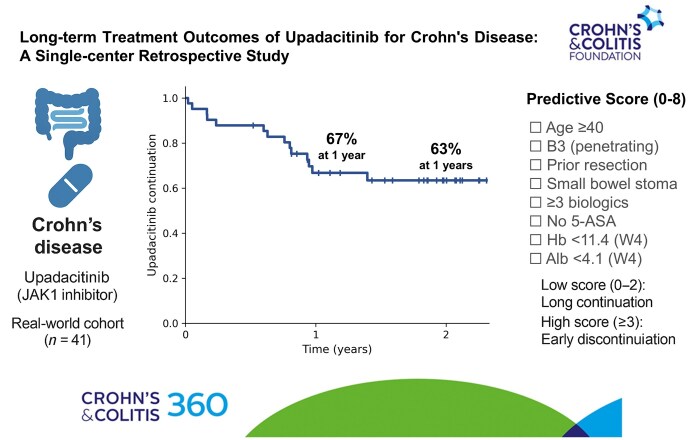

## Introduction

Crohn’s disease is a chronic inflammatory bowel disease characterized by transmural inflammation affecting any gastrointestinal tract segment.^1^ With the introduction of biologic therapies and small-molecule inhibitors, its therapeutic landscape has evolved substantially.^2^ Janus kinase (JAK) inhibitors represent a novel therapeutic class that targets inflammation-associated intracellular signaling pathways.^3^

Upadacitinib, a selective JAK1 inhibitor, has demonstrated efficacy for moderate to severe Crohn’s disease in clinical trials.^4^^,^^5^ In June 2023, upadacitinib became Japan’s sole approved JAK inhibitor for Crohn’s disease. Pivotal phase 3 clinical trials such as U-EXCEL and U-EXCEED revealed that upadacitinib can effectively induce and maintain clinical remission in patients with Crohn’s disease.^5^

Although randomized controlled trials demonstrate upadacitinib’s efficacy under controlled conditions, real-world studies are essential to evaluate its performance in heterogeneous clinical populations, including those with multiple prior treatment failures.^6^ Several real-world studies have reported short-term outcomes of upadacitinib in Crohn’s disease. Chugh et al.^7^ reported outcomes from a multicenter cohort, and Danso et al.^8^ described real-world effectiveness in a European population. However, long-term treatment outcomes of upadacitinib in Crohn’s disease remain largely unknown.

Therefore, this single-center retrospective study aimed to evaluate the long-term continuation rates of upadacitinib in patients with Crohn’s disease, identify factors associated with treatment discontinuation, develop a predictive scoring system for treatment continuation, and assess upadacitinib’s effects on perianal fistulas, endoscopic outcomes, and adverse events.

## Methods

### Study design and patients

This single-center, retrospective observational study included consecutive patients with established Crohn’s disease who initiated upadacitinib therapy between July 2023 and April 2025 at the Inflammatory Bowel Disease Center of Sapporo Higashi Tokushukai Hospital, Japan. The diagnosis of Crohn’s disease was based on clinical, endoscopic, histological, and radiological criteria according to established guidelines. All patients were treated at our specialized IBD center. The IBD Center is staffed by 9 gastroenterologists: 4 IBD specialists (3 with more than 20 years of IBD experience and 1 with more than 10 years of experience) and 5 general gastroenterologists. Treatment decisions were made collaboratively following institutional protocols under the supervision of IBD specialists. All patients received upadacitinib at a 45 mg dose once daily for 12 weeks as an induction therapy, followed by 15 or 30 mg once daily as a maintenance therapy based on clinical response at week 12. In Japan, concomitant use of immunomodulators (azathioprine or methotrexate) with upadacitinib is not covered by the national health insurance system and is therefore not permitted in clinical practice.

### Outcomes

The primary outcome was the cumulative continuation rate of upadacitinib treatment at 1 and 2 years from treatment initiation. Cessation of upadacitinib for any reason, including lack of efficacy, adverse events, or patient preference, defined treatment discontinuation.

Secondary outcomes included the following: (1) factors associated with treatment discontinuation identified using univariate analysis; (2) a predictive scoring system for long-term treatment continuation; (3) perianal draining fistula outcomes within 24 weeks of treatment initiation; (4) endoscopic response as measured by the Simple Endoscopic Score for Crohn’s Disease (SES-CD)^9^; and (5) adverse events.

### Data collection

Demographic and clinical data were extracted from patients’ electronic medical records. Baseline characteristics included age, sex, disease duration, body mass index (BMI), smoking status, disease location (Montreal classification L1-L3), disease behavior (Montreal classification B1-B3), intestinal resection history, stoma presence, perianal draining fistulas, extraintestinal manifestations, prior biologic exposure, and concomitant medications. Laboratory parameters consisted of Crohn’s Disease Activity Index (CDAI), white blood cell (WBC) count, hemoglobin, platelet count, albumin, C-reactive protein (CRP), leucine-rich alpha-2-glycoprotein (LRG), and fecal calprotectin (Fcal). LRG is a serum biomarker that has been validated and is widely used in Japan for monitoring IBD disease activity. It reflects intestinal inflammation and is covered by Japanese national health insurance for IBD monitoring. Unlike CRP, LRG may be elevated in patients with active IBD even when CRP is normal. Endoscopic disease activity was assessed using SES-CD for those who underwent colonoscopy.^9^

### Statistical analysis

Continuous variables are expressed as median (range) and categorical variables as number (percentage). Treatment continuation rates were estimated using the Kaplan-Meier method. For the univariate analysis, the log-rank test was used to identify factors associated with treatment discontinuation. Univariate Cox proportional hazards regression was used to calculate hazard ratios (HRs) with 95% confidence intervals (CIs). Continuous variables were dichotomized at the median value. For the predictive scoring system, factors significantly associated with treatment discontinuation in the univariate analysis were assigned 1 point each, and patients were stratified by the total score. Paired endoscopic data were compared using the Wilcoxon signed-rank test. Furthermore, adverse event rates were calculated as incidence rates per 100 patient-years with Poisson 95% confidence intervals (CIs). A *P-*value below .05 indicated statistical significance. All statistical data were analyzed using EZR (Saitama Medical Center, Jichi Medical University, Saitama, Japan), a graphical user interface for R (The R Foundation for Statistical Computing, Vienna, Austria).

## Results

### Patient characteristics

This study included 41 patients with Crohn’s disease who initiated upadacitinib during the study period. Table 1 summarizes the baseline characteristics. The median age was 41 years (range, 18-70 years), and 32 (78.0%) of them were male. The median disease duration was 220 months (range, 9-465 months). Most of the patients had ileocolonic disease (L3; 78.0%), and the disease behavior was predominantly stricturing (B2; 46.3%) or penetrating (B3; 41.5%). Of all patients, 27 (65.9%) had prior intestinal resection, and 8 (19.5%) developed a stoma. At baseline, 5 (12.2%) presented with perianal draining fistulas. Six patients (14.6%) had extraintestinal manifestations at baseline, all of which were peripheral arthritis.

**Table 1:** Baseline characteristics of patients.

Characteristic (*N *= 41)	*n* (%) or median (range)
**Age, years**	41 (18-70)
**Male sex**	32 (78.0)
**Disease duration, months**	220 (9-465)
**BMI, kg/m²**	21.2 (15.4-37.9)
**Active smoker**	14 (34.1)
** Disease location (Montreal classification)**	
** L1 (ileal)**	8 (19.5)
** L2 (colonic)**	1 (2.4)
** L3 (ileocolonic)**	32 (78.0)
** Disease behavior (Montreal classification)**	
** B1 (inflammatory)**	5 (12.2)
** B2 (stricturing)**	19 (46.3)
** B3 (penetrating)**	17 (41.5)
**Prior intestinal resection**	27 (65.9)
**Stoma**	
** None**	33 (80.5)
** Small bowel**	5 (12.2)
** Large bowel**	3 (7.3)
**Perianal draining fistula**	5 (12.2)
**Extraintestinal manifestations**	6 (14.6)
**Prior advanced therapy exposure**	
** Infliximab**	23 (56.1)
** Adalimumab**	23 (56.1)
** Vedolizumab**	14 (34.1)
** Ustekinumab**	27 (65.9)
** Risankizumab**	3 (7.3)
** Biologic-naive**	5 (12.2)
** 1 biologic**	11 (26.8)
** 2 biologics**	11 (26.8)
** ≥3 biologics**	15 (36.6)
**Baseline disease activity**	
** CDAI**	147 (0-399)
** WBC, /µL**	6170 (2910-13 780)
** Hemoglobin, g/dL**	11.8 (6.2-17.0)
** Platelet, ×10^4^/µL**	28.1 (12.6-156.1)
** Albumin, g/dL**	3.7 (1.8-5.0)
** CRP, mg/dL**	0.34 (0.01-7.37)
** LRG, µg/mL (*n *= 35)**	21.9 (9.6-62.9)
** Fcal, mg/kg (*n *= 7)**	360 (91.5-2635)
** SES-CD (*n *= 27)**	8.0 (0-29)
**Concomitant medications**	
** 5-ASA**	28 (68.3)
** Enteral nutrition (≥900 kcal/day)**	14 (34.1)
** Budesonide**	8 (19.5)
**Maintenance dose of upadacitinib**	
** 30 mg**	32 (86.5)
** 15 mg**	5 (13.5)
**Herpes zoster vaccination**	0 (0)

Abbreviations: 5-ASA, 5-aminosalicylic acid; BMI, body mass index; CDAI, Crohn’s Disease Activity Index; CRP, C-reactive protein; Fcal, fecal calprotectin; LRG, leucine-rich alpha-2-glycoprotein; SES-CD, Simple Endoscopic Score for Crohn’s Disease; WBC, white blood cell.

**Figure 1:**
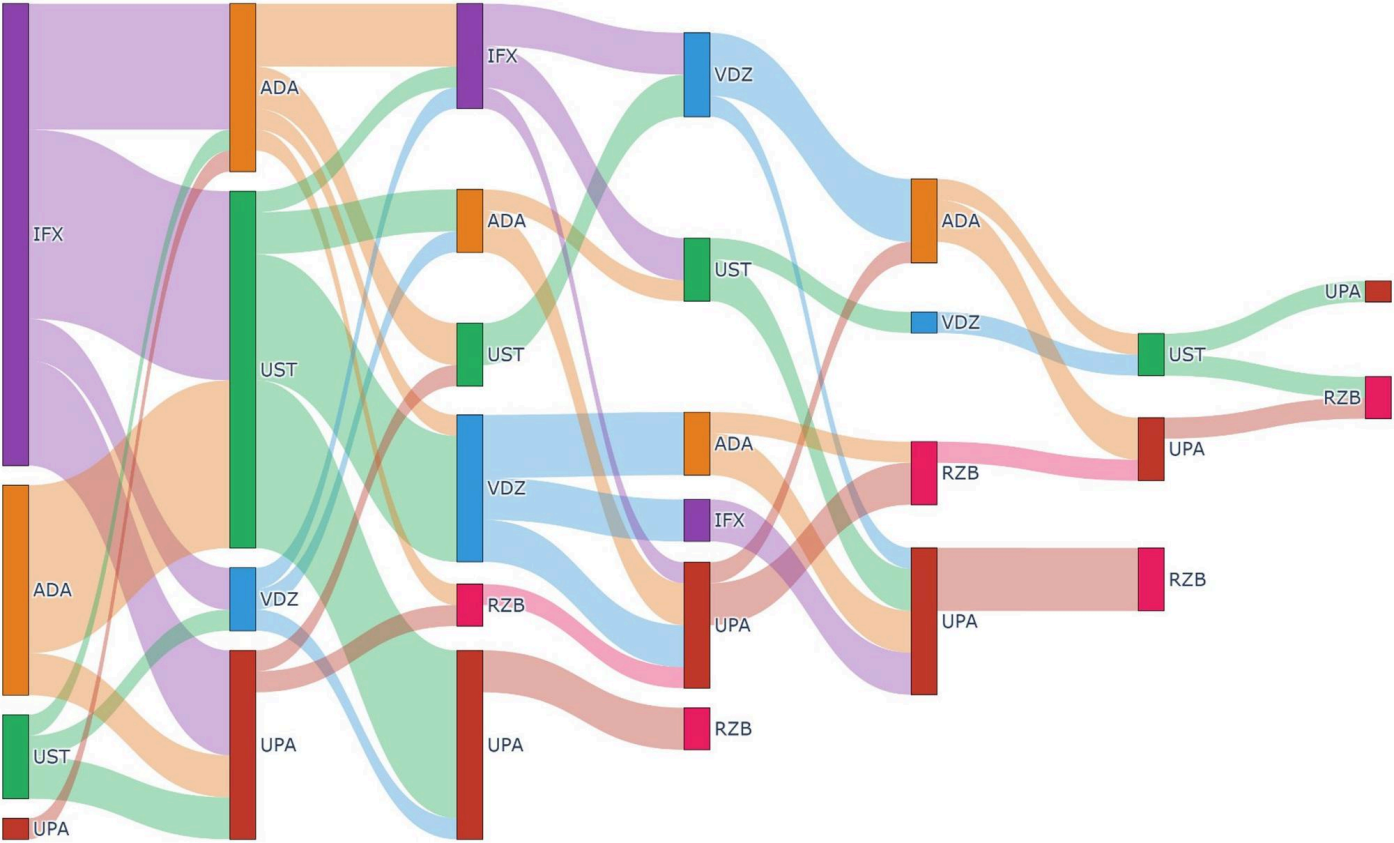
Treatment sequence flow diagram (Sankey diagram). Treatment sequence flow diagram (Sankey diagram) showing the utilization patterns of upadacitinib in the study cohort. This diagram illustrates the sequence of advanced therapies before and after upadacitinib initiation. IFX, infliximab; ADA, adalimumab; UST, ustekinumab; VDZ, vedolizumab; UPA, upadacitinib; RZB, risankizumab.

Regarding prior biologic exposure, only 5 (12.2%) patients were biologic-naive. Ustekinumab (65.9%) was the most commonly used prior biologic, while risankizumab (7.3%) was the least. Moreover, 15 (36.6%) patients had been exposed to 3 or more prior biologics. Figure 1 illustrates the flow of treatment sequences.

The median values for baseline characteristics were as follows: CDAI, 147 (range, 0-399); WBC count, 6170/µL (2910-13 780/µL); hemoglobin, 11.8 (6.2-17.0) g/dL; platelet count, 28.1 × 10^4^/µL (12.6-156.1 × 10^4^/µL); albumin, 3.7 (1.8-5.0) g/dL; CRP, 0.34 (0.01-7.37) mg/dL; LRG, 21.9 (9.6-62.9) µg/mL; and Fcal, 360 (91.5-2635). The median SES-CD in 27 patients who underwent baseline colonoscopy was 8.0 (0-29). Concomitant medications at baseline were 5-aminosalicylic acid (5-ASA), enteral nutrition (≥900 kcal/day), and budesonide in 28 (68.3%), 14 (34.1%), and 8 (19.5%) patients, respectively. None of the 41 patients had received the recombinant herpes zoster vaccine (Shingrix) prior to upadacitinib initiation, reflecting the limited availability and high cost of Shingrix in Japan, where it is not covered by national health insurance for this indication.

### Treatment continuation rates

**Figure 2:**
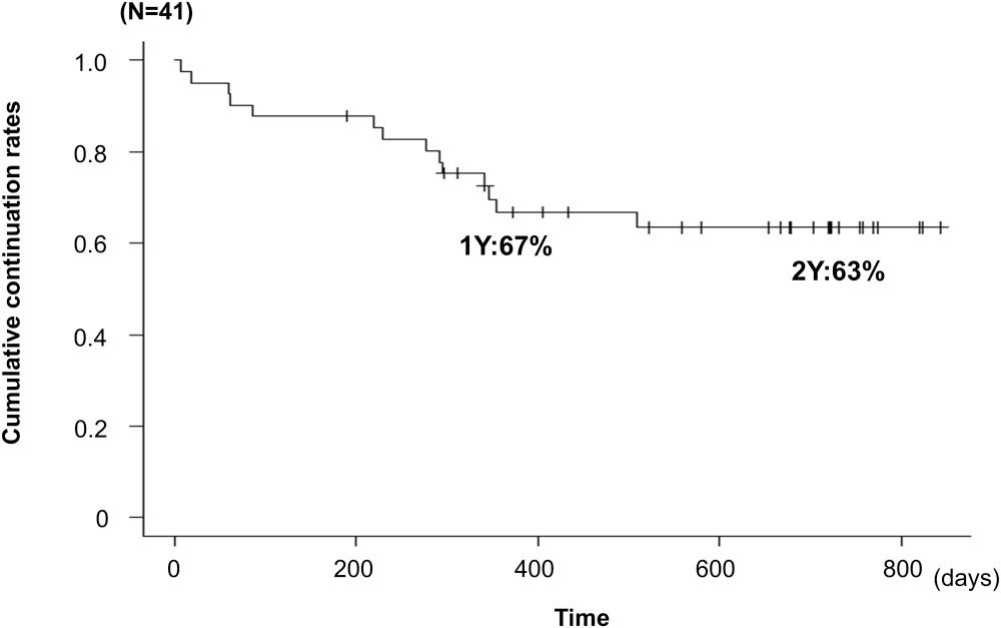
Cumulative continuation rates of upadacitinib treatment. Kaplan-Meier curve showing the cumulative continuation rates of upadacitinib treatment in 41 patients with Crohn’s disease. The 1-year and 2-year continuation rates were 67% and 63%, respectively.

The cumulative continuation rates of upadacitinib treatment were 67% at 1 year and 63% at 2 years (Figure 2). Of the 37 patients who transitioned to maintenance therapy, 32 (86.5%) continued on 30 mg and 5 (13.5%) were reduced to 15 mg. Four patients (9.8%) discontinued during the induction phase. Of the 14 patients who discontinued treatment, 9 (64.3%) discontinued due to primary non-response, 3 (21.4%) due to secondary loss of response, and 2 (14.3%) due to adverse events (1 deep vein thrombosis and 1 persistent acne). No patients discontinued due to patient preference alone.

### Factors associated with treatment discontinuation

Univariate analysis identified several factors significantly associated with treatment discontinuation (Table 2). Patient-related factors included age ≥ 40 years (*P *= .038), penetrating disease behavior (B3) (*P *= .026), prior intestinal resection (*P *= .046), and small-bowel stoma presence (*P *< .001). Of the 8 patients with stomas, 5 had small bowel stomas and 3 had colostomies. Treatment discontinuation was more common in those with small bowel stomas (4/5, 80%) compared to colostomies (1/3, 33%), although the numbers are too small for statistical comparison. Treatment history factors were prior exposure to 3 or more biologics (*P *= .004) and absence of concomitant 5-ASA therapy (*P *= .036).

**Table 2:** Univariate analysis of factors associated with upadacitinib treatment continuation.

Factor	HR	95% CI	*P*-value
**Age ≥40 years**	2.38	1.04-5.45	.038
**Male sex**	0.72	0.25-2.06	.42
**Disease duration ≥220 months**	1.32	0.58-3.00	.47
**BMI ≥21.2 kg/m²**	1.25	0.55-2.84	.54
**Disease location L1 (ileum)**	0.77	0.26-2.30	.55
**Disease behavior B1 (inflammatory)**	0.68	0.16-2.90	.45
**Disease behavior B3 (penetrating)**	2.56	1.08-6.07	.026
**Perianal draining fistula**	1.12	0.33-3.80	.82
**Active smoker**	1.98	0.87-4.52	.073
**Prior intestinal resection**	2.31	1.01-5.29	.046
**Small bowel stoma**	5.42	2.01-14.6	<.001
**Biologic-naive**	0.78	0.18-3.38	.65
**≥3 prior biologics**	3.21	1.42-7.26	.004
**Baseline CDAI ≥151**	0.87	0.38-1.99	.68
**Baseline WBC ≥6170/µL**	1.68	0.74-3.82	.166
**Baseline Hb ≥11.8 g/dL**	0.48	0.21-1.10	.073
**Baseline Plt ≥28.1 × 10^4^/µL**	1.38	0.60-3.17	.41
**Baseline Albumin ≥3.7 g/dL**	1.06	0.47-2.39	.87
**Baseline CRP <0.34 mg/dL**	2.52	1.09-5.83	.028
**Baseline SES-CD <8**	3.28	1.33-8.09	.049
**Week 4 CDAI ≥151**	1.35	0.59-3.08	.45
**Week 4 WBC ≥6350/µL**	1.52	0.67-3.45	.29
**Week 4 Hb <11.4 g/dL**	4.85	2.03-11.6	<.001
**Week 4 Plt ≥30.1 × 10^4^/µL**	1.48	0.65-3.37	.32
**Week 4 albumin <4.1 g/dL**	3.42	1.38-8.48	.006
**Week 4 CRP ≥0.11 mg/dL**	1.18	0.52-2.68	.69
**Concomitant 5-ASA**	0.42	0.18-0.98	.036
**Enteral nutrition (≥900 kcal/day)**	0.46	0.20-1.06	.067
**Budesonide**	2.08	0.91-4.76	.066

Abbreviations: 5-ASA, 5-aminosalicylic acid; BMI, body mass index; CDAI, Crohn’s Disease Activity Index; CRP, C-reactive protein; Fcal, fecal calprotectin; Hb, hemoglobin; LRG, leucine-rich alpha-2-glycoprotein; Plt, platelet; SES-CD, Simple Endoscopic Score for Crohn’s Disease; WBC, white blood cell.

Regarding disease activity parameters, lower baseline inflammation was paradoxically associated with worse continuation rates. In particular, baseline CRP < 0.34 mg/dL (*P *= .028) and baseline SES-CD < 8 (*P *= .049) were both significantly related to treatment discontinuation. Early biomarkers at week 4, particularly hemoglobin < 11.4 g/dL (*P *< .001) and albumin < 4.1 g/dL (*P *= .006), were also predictive of treatment discontinuation.

### Predictive scoring system

A predictive scoring system for long-term treatment continuation was developed using the following 8 factors that were significantly associated with treatment discontinuation in the univariate analysis: age ≥ 40 years, penetrating disease behavior (B3), prior intestinal resection, small-bowel stoma presence, prior exposure to 3 or more biologics, absence of concomitant 5-ASA therapy, week 4 hemoglobin < 11.4 g/dL, and week 4 albumin < 4.1 g/dL. Each factor was assigned one point (Table 3).

**Table 3:** Predictive scoring system for long-term treatment continuation.

Factor	Score
**Age ≥ 40 years**	1
**Penetrating disease behavior (B3)**	1
**Prior intestinal resection**	1
**Small-bowel stoma**	1
**Prior exposure to ≥3 biologics**	1
**No concomitant 5-ASA**	1
**Week 4 hemoglobin <11.4 g/dL**	1
**Week 4 albumin <4.1 g/dL**	1
**Total**	0-8

**Figure 3:**
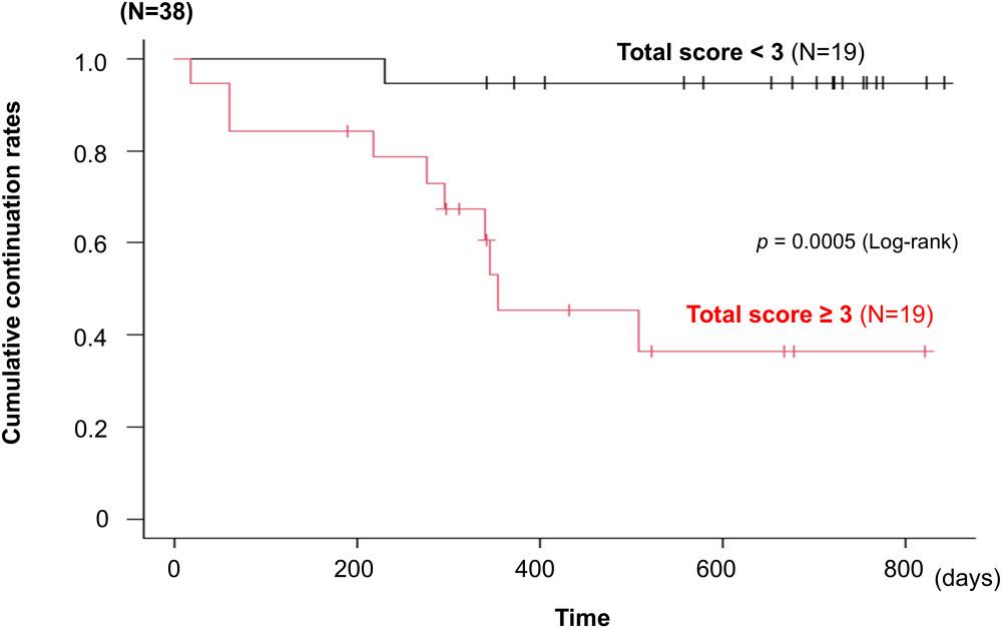
Cumulative continuation rates between patients with low and high predictive scores. Kaplan-Meier curves comparing the cumulative continuation rates between patients with low (0-2 points) and high (≥3 points) predictive scores. The low-score group demonstrated significantly better continuation rates than the high-score group (*P* = .0005, log-rank test).

Patients were stratified into low-score (0-2 points) and high-score (3-6 points) groups. In the analysis of 38 patients with complete scoring data, the treatment continuation rates were significantly better in the low-score group than in the high-score group (*P *= .0005, log-rank test; Figure 3). The median treatment duration was longer in the low-score group than in the high-score group (703 days vs. 341 days).

### Perianal draining fistula outcomes

Among the 5 patients with perianal draining fistulas at baseline, 2 (40%) achieved fistula closure within 24 weeks (Table 4). Both patients had lower inflammatory burden (lower CRP levels) and less extensive exposure to prior biologics than nonresponders.

**Table 4:** Perianal draining fistula outcomes.

Case	Sex	Location	Behavior	Smoker	Prior biologics	Perianal surgery	CDAI	CRP	SES-CD	Closure ≤24 wk
**1**	M	L3	B3	Yes	3	Yes	196	7.37	15	No
**2**	M	L3	B3	No	4	Yes	Stoma	2.51	NA	No
**3**	F	L3	B2	Yes	4	Yes	171	0.25	12	No
**4**	M	L1	B2	No	1	No	79	0.37	NA	Yes
**5**	M	L3	B2	No	0	No	42	1.02	6	Yes

Abbreviations: CDAI, Crohn’s Disease Activity Index; CRP, C-reactive protein; wk, weeks.

### Endoscopic outcomes

**Figure 4:**
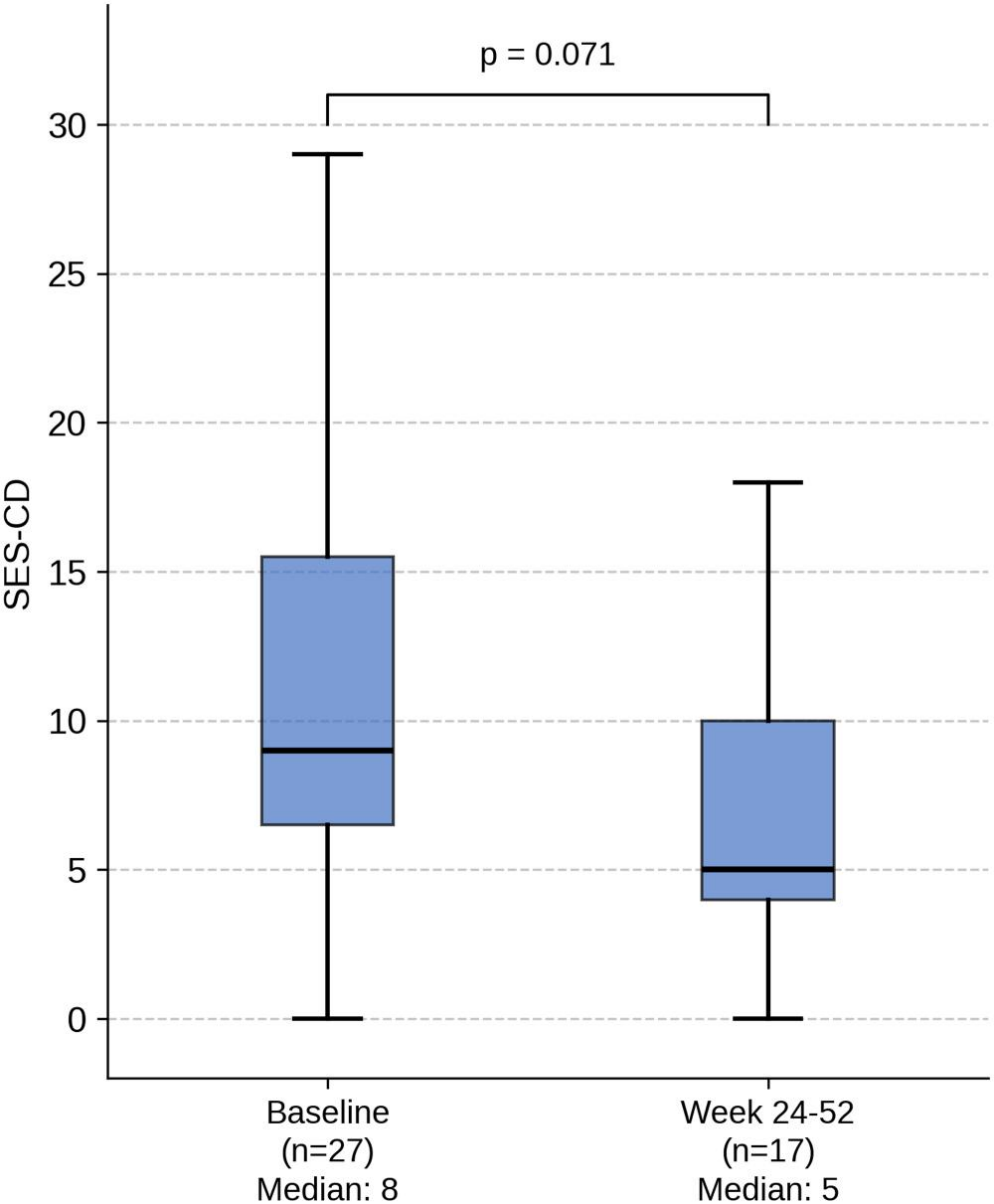
Changes in SES-CD. Box plot showing SES-CD at baseline (median 8, *n* = 27) and week 24-52 (median 5, *n* = 17). The change did not reach statistical significance (*P* = .071, Wilcoxon signed-rank test).

The endoscopic data of 27 patients with colonoscopy experience were evaluated at baseline and between weeks 24 and 52 in 17 patients. The median SES-CD decreased from 8.0 at baseline to 5.0 at follow-up (Figure 4), although this difference was not statistically significant (*P *= .071, Wilcoxon signed-rank test).

### Adverse events

**Figure 5:**
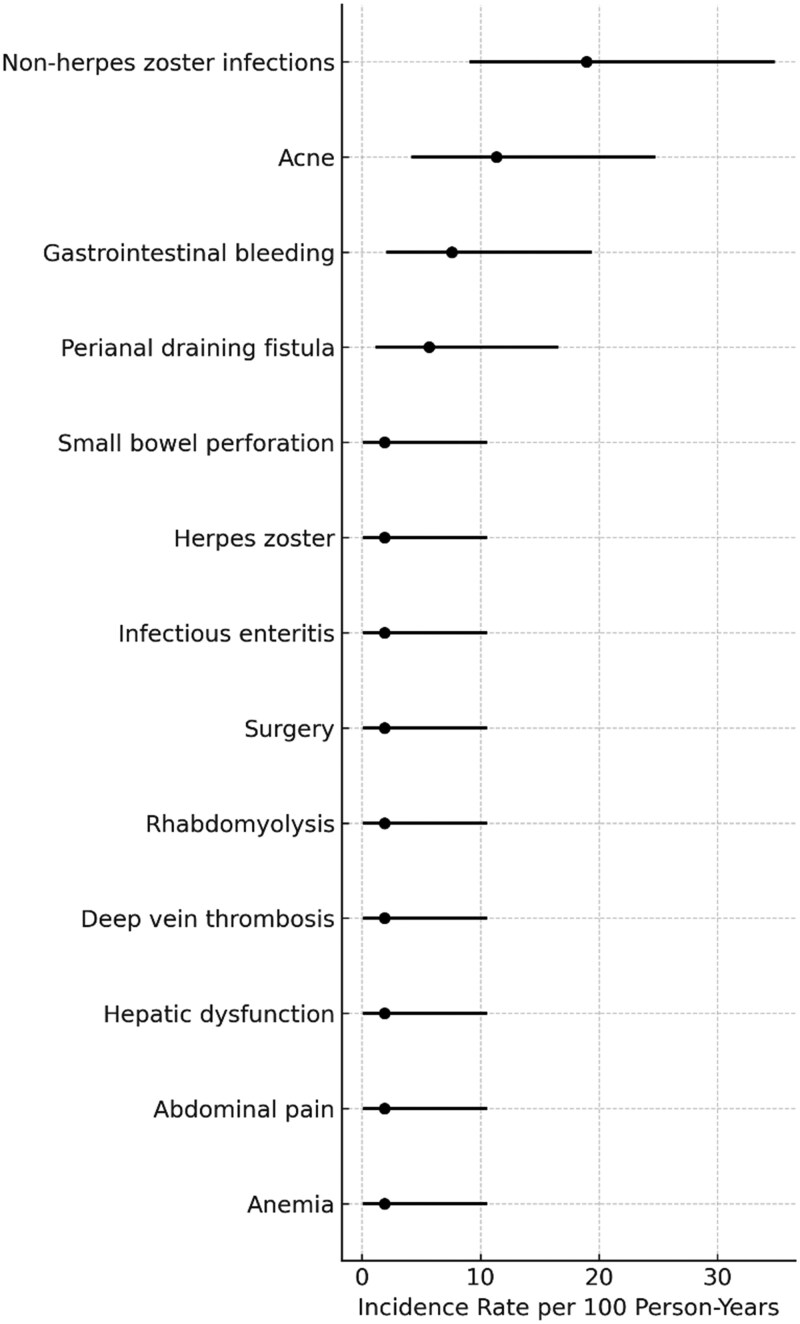
Incidence rates of adverse events. Incidence rates of adverse events during upadacitinib treatment. Error bars represent 95% confidence intervals calculated using the Poisson distribution. Rates are expressed per 100 patient-years.

During the total observation period of 52.8 patient-years, various adverse events were recorded (Table 5, Figure 5). The most common adverse events were infections (excluding herpes zoster) (incidence rate, 18.94 per 100 patient-years; 95% CI, 9.08-34.83) and acne (11.36 per 100 patient-years; 95% CI, 4.17-24.73). Furthermore, 4 patients experienced gastrointestinal bleeding (7.58 per 100 patient-years; 95% CI, 2.06-19.40), and 3 developed new perianal draining fistulas (5.68 per 100 patient-years; 95% CI, 1.17-16.60).

**Table 5:** Adverse events during upadacitinib treatment.

Adverse event	Events (*n*)	IR (100 PY)	95% CI (100 PY)
**Infections (excluding herpes zoster)**	10	18.94	9.08-34.83
**Acne**	6	11.36	4.17-24.73
**Gastrointestinal bleeding**	4	7.58	2.06-19.40
**New perianal draining fistula**	3	5.68	1.17-16.60
**Small-bowel perforation**	1	1.89	0.05-10.55
**Herpes zoster**	1	1.89	0.05-10.55
**Infectious enteritis**	1	1.89	0.05-10.55
**Surgery**	1	1.89	0.05-10.55
**Rhabdomyolysis**	1	1.89	0.05-10.55
**Deep vein thrombosis**	1	1.89	0.05-10.55
**Liver dysfunction**	1	1.89	0.05-10.55
**Abdominal pain**	1	1.89	0.05-10.55
**Anemia**	1	1.89	0.05-10.55

Abbreviations: CI, confidence interval; IR, incidence rate; PY, patient-years.

Adverse events included single cases of small bowel perforation, herpes zoster, infectious enteritis, surgery, rhabdomyolysis, deep vein thrombosis, liver dysfunction, abdominal pain, and anemia (each: 1.89 per 100 patient-years; 95% CI, 0.05-10.55). The patient who developed herpes zoster had not been vaccinated. Despite the absence of herpes zoster vaccination in our cohort, only one case (2.4%) occurred during follow-up.

## Discussion

This single-center retrospective study provides real-world evidence on the long-term treatment outcomes of upadacitinib in patients with Crohn’s disease. Approximately two-thirds of these patients continued upadacitinib treatment at 1 year, with similar rates maintained at 2 years. In this study, multiple predictive factors for treatment discontinuation were identified. A scoring system was also developed to identify which patients are most likely to benefit from long-term upadacitinib therapy.

Chugh et al.^7^ reported similar short-term outcomes in their multicenter cohort study. Danso et al.^8^ described real-world effectiveness in a European population. Our study extends these findings by providing 2-year follow-up data from a Japanese population and developing a predictive scoring system for treatment continuation. A Japanese multicenter real-world study reported high continuation rates of upadacitinib in 33 patients with Crohn’s disease.^10^ In the present study, the relatively high proportion of patients with prior biologic experience (87.8%) shows that upadacitinib is often a later-line therapy in clinical practice. Despite this challenging patient population, the 67% continuation rate at 1 year suggests that upadacitinib offers a meaningful clinical benefit in numerous patients.

Several factors emerged as predictors of treatment discontinuation. The association of older age with worse outcomes may reflect longstanding disease with irreversible structural damage or altered drug metabolism.^6^ Factors such as penetrating disease behavior and prior intestinal resection likely indicate more severe disease phenotypes that may be less responsive to medical therapy. Furthermore, the presence of a small-bowel stoma was strongly related to treatment discontinuation, possibly reflecting patients with a particularly refractory disease or an inadequate bowel length for drug absorption.

The association of prior exposure to multiple biologics with treatment discontinuation supports the concept of reduced efficacy with successive therapies in inflammatory bowel disease.^11^ Interestingly, concomitant 5-ASA therapy was linked to better outcomes in the present study, although this may reflect a marker of less severe disease rather than a direct therapeutic benefit of 5-ASA, as 5-ASA has limited evidence for efficacy in Crohn’s disease according to international guidelines.

In the early biomarker analysis, week 4 hemoglobin and albumin levels were strong predictors of long-term outcomes. Thus, early assessment of nutritional and inflammatory status may help identify patients needing treatment intensification or switching.^12^^,^^13^ Given the rapid onset of action of JAK inhibitors, early response assessment is particularly relevant.^3^

Interestingly, our study revealed a paradoxical finding regarding baseline disease activity: patients with lower baseline inflammation (CRP < 0.34 mg/dL and SES-CD < 8) had worse continuation rates. This counterintuitive finding may reflect patients with predominantly fibrostenotic rather than inflammatory disease, in whom JAK inhibition targeting inflammatory pathways would be less effective. The association of lower baseline inflammation with treatment discontinuation was primarily due to perceived primary non-response rather than adverse events, supporting this hypothesis. These findings highlight the complexity of selecting treatments in heterogeneous Crohn’s disease populations and prompt the need for further investigation in larger cohorts.

The predictive scoring system developed in this study is a practical tool for stratifying risks. Patients with low scores (0-2 points) continued the treatment substantially longer than those with high scores (≥3 points). Equal weighting was chosen for clinical simplicity and ease of use in practice, as weighted scoring systems often provide only marginal improvements in predictive accuracy while significantly increasing complexity. The threshold of 0-2 versus ≥3 was determined by maximizing the separation in continuation curves. Baseline CRP and SES-CD were intentionally excluded from the scoring system because their paradoxical association (lower values associated with worse outcomes) would make clinical interpretation counterintuitive and potentially confusing in a bedside scoring tool. Although this scoring system requires external validation, it may help guide treatment decisions and patient counseling.

In this study, upadacitinib demonstrated limited efficacy in a small number of patients who developed perianal fistulas, with closure achieved in 2 of 5 patients. This finding aligns with the limited data available on JAK inhibitor efficacy for perianal disease, underscoring the need for larger studies that specifically address this important clinical manifestation.

The safety profile observed in our study generally agrees with the known adverse event profile of upadacitinib.^4^^,^^5^ Infections were the most common adverse events, followed by acne, a known effect of upadacitinib. However, the occurrence of single cases of deep vein thrombosis and rhabdomyolysis warrants attention. The follow-up duration (median approximately 14 months) may be insufficient to capture rare long-term safety signals such as malignancy and major adverse cardiovascular events, which require years of observation.

This study has several limitations. First, the single-center retrospective design limits the generalizability of the study findings and introduces potential selection bias. Second, the relatively small sample size, particularly for subgroup analyses, limits statistical power and precludes multivariable analysis (typically requiring 10-15 events per predictor variable). Third, our cohort was predominantly male (78%), which may limit generalizability to female patients. Fourth, baseline endoscopic assessment was available in only 27 of 41 patients (65.9%), which limits the interpretation of endoscopic outcomes. Fifth, the relatively low median baseline SES-CD (8) may reflect a population with less severe endoscopic disease, limiting generalizability to patients with more severe endoscopic inflammation. Sixth, the low proportion of biologic-naive patients prevents conclusions regarding first-line use. Seventh, treatment selection was not randomized, with unmeasured confounders likely influencing the outcomes, including physician treatment preferences, patient adherence, dietary factors, psychological factors, socioeconomic factors, and genetic factors. Finally, the follow-up duration may be insufficient to capture long-term safety signals. Nevertheless, our findings offer valuable real-world insights into long-term upadacitinib use for Crohn’s disease and may inform treatment guidelines and clinical decision-making.

## Conclusions

This real-world study demonstrates that upadacitinib provides sustained clinical benefit for approximately two-thirds of patients with Crohn’s disease at 1 year. Baseline patient characteristics and early biomarkers at week 4 may be helpful in predicting upadacitinib treatment continuation. In addition, the predictive scoring system developed in this study may help optimize patient selection for upadacitinib therapy. However, conducting multicenter prospective studies is warranted to validate these findings and further refine treatment algorithms for Crohn’s disease.

## Data Availability

Access to the data supporting the results of this study will be requested and reviewed with the principal investigator of this study through the corresponding author. The data are not available to the public due to privacy and ethical restrictions.
